# Luteolin triggers global changes in the microglial transcriptome leading to a unique anti-inflammatory and neuroprotective phenotype

**DOI:** 10.1186/1742-2094-7-3

**Published:** 2010-01-14

**Authors:** Konstantin Dirscherl, Marcus Karlstetter, Stefanie Ebert, Dominik Kraus, Julia Hlawatsch, Yana Walczak, Christoph Moehle, Rudolf Fuchshofer, Thomas Langmann

**Affiliations:** 1 Institute of Human Genetics, University of Regensburg, Franz-Josef-Strauss-Allee 11, 93053 Regensburg, Germany; 2 Center of Excellence for Fluorescent Bioanalytics, University of Regensburg, Josef-Engert-Str 9, 93053 Regensburg, Germany; 3 Institute of Human Anatomy and Embryology, University of Regensburg, Universitätsstr 31, 93053 Regensburg, Germany

## Abstract

**Background:**

Luteolin, a plant derived flavonoid, exerts a variety of pharmacological activities and anti-oxidant properties associated with its capacity to scavenge oxygen and nitrogen species. Luteolin also shows potent anti-inflammatory activities by inhibiting nuclear factor kappa B (NFkB) signaling in immune cells. To better understand the immuno-modulatory effects of this important flavonoid, we performed a genome-wide expression analysis in pro-inflammatory challenged microglia treated with luteolin and conducted a phenotypic and functional characterization.

**Methods:**

Resting and LPS-activated BV-2 microglia were treated with luteolin in various concentrations and mRNA levels of pro-inflammatory markers were determined. DNA microarray experiments and bioinformatic data mining were performed to capture global transcriptomic changes following luteolin stimulation of microglia. Extensive qRT-PCR analyses were carried out for an independent confirmation of newly identified luteolin-regulated transcripts. The activation state of luteolin-treated microglia was assessed by morphological characterization. Microglia-mediated neurotoxicity was assessed by quantifying secreted nitric oxide levels and apoptosis of 661W photoreceptors cultured in microglia-conditioned medium.

**Results:**

Luteolin dose-dependently suppressed pro-inflammatory marker expression in LPS-activated microglia and triggered global changes in the microglial transcriptome with more than 50 differentially expressed transcripts. Pro-inflammatory and pro-apoptotic gene expression was effectively blocked by luteolin. In contrast, mRNA levels of genes related to anti-oxidant metabolism, phagocytic uptake, ramification, and chemotaxis were significantly induced. Luteolin treatment had a major effect on microglial morphology leading to ramification of formerly amoeboid cells associated with the formation of long filopodia. When co-incubated with luteolin, LPS-activated microglia showed strongly reduced NO secretion and significantly decreased neurotoxicity on 661W photoreceptor cultures.

**Conclusions:**

Our findings confirm the inhibitory effects of luteolin on pro-inflammatory cytokine expression in microglia. Moreover, our transcriptomic data suggest that this flavonoid is a potent modulator of microglial activation and affects several signaling pathways leading to a unique phenotype with anti-inflammatory, anti-oxidative, and neuroprotective characteristics. With the identification of several novel luteolin-regulated genes, our findings provide a molecular basis to understand the versatile effects of luteolin on microglial homeostasis. The data also suggest that luteolin could be a promising candidate to develop immuno-modulatory and neuroprotective therapies for the treatment of neurodegenerative disorders.

## Background

Microglia, the resident macrophages of the nervous system, have important roles in immune regulation [1,2] and neuronal homeostasis [3,4]. Microglia belong to the mononuclear phagocyte system but their special localization in the fragile neuronal environment and their morphological features clearly distinguish them from other peripheral macrophages [5]. Ramified microglia perform a very active and continous surveillance function with their long protrusions [6,7]. They receive permanent tonic inhibitory inputs from neurons to prevent microglial neurotoxicity [8,9]. Loss of microglia-neuron cross-talk [10], local danger signals such as extracellular ATP [11], or neurotransmitter gradients [12] rapidly lead to a functional transformation of ramified microglia with a variety of effector functions.

Microglia activation is a protective mechanism regulating tissue repair and recovery in the early phase of neurodegeneration [4]. However, excessive or sustained activation of microglia often contributes to acute and chronic neuro-inflammatory responses in the brain and the retina [2]. Activated microglia in the vicinity of degenerating neurons have been identified in a broad spectrum of neurodegenerative disorders including Alzheimer's disease [13], Parkinson's disease [14], amyotrophic lateral sclerosis [15], multiple sclerosis [16], and inherited photoreceptor dystrophies [17,18].

Macrophage heterogeneity and plasticity is very large and the set of marker combinations and sub-populations is essentially infinite [19]. To define a simplified conceptual framework, classification into polarized functional categories, called M1 and M2 macrophages has been proposed [20,21]. M1 or "classically activated" macrophages produce high levels of oxidative metabolites and pro-inflammatory cytokines but also cause damage to healthy tissue as side effect [22]. M2 or "alternatively activated" macrophages promote tissue remodeling and generally suppress destructive immune reactions. Informations on microglial subsets in the nervous system are relatively scarce compared to other tissue macrophages. Nevertheless, recent findings from *in vitro *cultures of the murine microglial cell line MMGT12 [23] and hippocampal microglia from the PS1xAPP Alzheimer's mouse model [24] implicate that microglia have the ability to differentiate into M1 and M2 polarized phenotypes. A co-existence of neurotoxic M1 microglia and regenerative M2 microglia has been recently documented in the injured mouse spinal cord [25]. Microarray-based quantitation of M1 and M2 markers as well as functional tests on axonal regrowth after injury demonstrated that a transient anti-inflammatory and neuroprotective M2 response was rapidly overwhelmed by a neurotoxic M1 microglial response [25]. A similar but age-dependent switch from alternative to classical activation was shown in PS1xAPP Alzheimer's mice [24], indicating a common phenomenon in neurodegenerative disorders. Compounds that induce the switch of microglia from inflammatory M1 type to anti-inflammatory M2 type could therefore be a potential therapeutic agent to attenuate neuronal inflammation and boost neuronal recovery [26].

Several anti-inflammatory drugs have been shown to diminish neuroinflammation, but only a few direct functional effects on microglial activity have been elucidated [27]. Among the naturally occuring immuno-modulators, the flavonoid luteolin (3',4',5,7-tetrahydroxyflavone), abundant in parsley, green pepper, celery, perilla leaf, and chamomile tea, exerts prominent anti-inflammatory and anti-oxidant activities [28]. Luteolin suppressed pro-inflammatory cytokine production in macrophages by blocking nuclear factor kappa B (NFkB) and activator protein 1 (AP1) signaling pathways [29] and inhibited the production of nitric oxide [30] and pro-inflammatory eicosanoids [31]. Luteolin also diminshed the release of Tnf and superoxide anions in LPS or interferon-γ treated microglial cell cultures [32,33] and reduced the LPS-induced Il6 production in brain microglia *in vivo *[34].

Although the inhibitory function of luteolin on NFkB and a few selected cytokines is well documented in macrophages, a genome-wide search for further molecular targets in microglia has not yet been published. Furthermore, the immuno-modulatory effects of luteolin related to the stimulation of distinct functional microglial phenotypes has not been investigated before. Therefore, this study investigated the global transcriptomic effects of luteolin at near physiological concentrations [35] alone or in combination with LPS in pure BV-2 microglial cultures. We further validated the luteolin-regulated expression of novel pro- and anti-inflammtory microglial transcripts, analyzed microglial morphology, and studied the consequences of microglia-conditioned media for photoreceptor viability.

## Methods

### Reagents

Luteolin (3',4',5,7-tetrahydroxyflavone) and *E. coli *0111:B4 lipopolysaccharide were purchased from Sigma Aldrich (Steinheim, Germany). Luteolin was dissolved in DMSO and added in concentrations that did not exceed 0.05% of the total volume in any of the cell culture experiments.

### Animals

C57BL/6 mice were purchased from Charles River Laboratories. Mice were kept in an air-conditioned barrier environment at constant temperature of 20-22°C on a 12-h light-dark schedule, and had free access to food and water. The health of the animals was regularly monitored, and all procedures were approved by the University of Regensburg animal rights committee and complied with the German Law on Animal Protection and the Institute for Laboratory Animal Research Guide for the Care and Use of Laboratory Animals, 1999.

### Cell culture

Brain microglia were isolated and cultured as described earlier [36]. BV-2 microglia-like cells were provided by Professor Ralph Lucius (Clinic of Neurology, Christian Albrechts University, Kiel, Germany). BV-2 cells were cultured in RPMI/5% FCS supplemented with 2 mM L-Glutamine and 195 nM β-mercaptoethanol. Primary brain microglia or BV-2 cells were stimulated with 10 ng/ml or 50 ng/ml LPS and various concentrations of luteolin for 24 h. 661W photoreceptor-like cells were a gift from Prof. Muayyad Al-Ubaidi (University of Illinois, Chicago, IL) and the culture conditions have been described elsewhere [36].

### Phalloidin staining

BV-2 cells were plated overnight on coverslips, fixed with 3.7% paraformaldehyde for 10 min at 37°C, permeabilized with 0.2% Triton X-100 for 5 min, blocked with 5% non-fat milk, 0.2% Triton X-100, and stained with DAPI for 10 min at room temperature (0.1 μg/ml in PBS, 4',6-diamidino-2-phenylindol, Molecular Probes). Filamentous actin was stained by addition of 1.5 μM TRITC-conjugated phalloidin (Sigma). The coverslips were mounted on microscopic glass slides and viewed with a Axioskop 2 fluorescence microscope equipped with an Eclipse digital analyzer (Carl Zeiss).

### NO measurement

NO concentrations were determined by measuring the amount of nitrite secreted by BV-2 cells into the culture medium using the Griess reagent system (Promega). 50 μl cell culture supernatant was collected and an equal volume of Griess reagent was added to each well. After incubation for 15 min at room temperature, the absorbance was read at 540 nm on a BMG FluoStar Optima plate reader (Labtech, Offenburg, Germany). The concentration of nitrite for each sample was calculated from a sodium nitrite standard curve.

### Apoptosis assay

Apoptotic cell death of 661W cells was determined with the Caspase-Glo^® ^3/7 Assay (Promega). Cells were lysed and incubated with a luminogenic caspase-3/7 substrate, which contains the tetrapeptide sequence DEVD. Luminescence was then generated by addition of recombinant luciferase and was proportional to the amount of caspase activity present. The luminescent signal was read on a BMG FluoStar Optima plate reader (Labtech, Offenburg, Germany). A blank reaction was used to measure background luminescence associated with the cell culture system and Caspase-Glo^® ^3/7 Reagent. The value for the blank reaction was subtracted from all experimental values. Negative control reactions were performed to determine the basal caspase activity of 661W cells.

### RNA isolation and reverse transcription

Total RNA was extracted from cultured microglia according to the manufacturer's instructions using the RNeasy Protect Mini Kit (Qiagen, Hilden, Germany). Purity and integrity of the RNA was assessed on the Agilent 2100 bioanalyzer with the RNA 6000 Nano LabChip^® ^reagent set (Agilent Technologies, Büblingen, Germany). The RNA was quantified spectrophotometrically and then stored at -80°C. First-strand cDNA synthesis was performed with RevertAid™ H Minus First Strand cDNA Synthesis Kit (Fermentas, St. Leon-Rot, Germany).

### DNA microarray analysis

Triplicate microarrays were carried out with three independent RNAs from non-stimulated BV-2 microglia or cultures treated for 24 h with 50 μM luteolin, 50 ng/ml LPS, or 50 μM LPS + 50 ng/ml LPS, respectively. Generation of double-stranded cDNA, preparation and labeling of cRNA, hybridization to Affymetrix 430 2.0 mouse genome arrays, washing, and scanning were performed according to the Affymetrix standard protocol. Minimum information about a microarray experiment (MIAME) criteria were met [37]. The microarray datasets of this study are publicly available at the National Center for Biotechnology Information Gene Expression Omnibus http://www.ncbi.nlm.nih.gov/geo/ as series record GSE18740.

### Bioinformatic data analysis

The Affymetrix Expression Console Software Version 1.0 was used to create summarized expression values (CHP-files) from 3' expression array feature intensities (CEL-files) using the Robust Multichip Analysis (RMA) algorithm. Integrative analysis of genome-wide expression activities from BV-2 cells was performed with the Gene Expression Dynamics Inspector (GEDI), a Matlab (Mathworks, Natick, MA) freeware program which uses self-organizing maps (SOMs) to translate high-dimensional data into a 2D mosaic [38]. Each tile of the mosaic represents an individual SOM cluster and is color-coded to represent high or low expression of the cluster's genes, thus identifying the underlying pattern.

Differentially regulated transcrips in 24 h luteolin stimulated versus non-treated and luteolin + LPS versus LPS-treated BV-2 cells, respectively, were retrieved with the Genomatix ChipInspector program (Genomatix Software GmbH, Munich, Germany), applying the Significance Analysis of Microarray (SAM) algorithm using a false-discovery rate of 0.1% and a minimum coverage of 3 independent probes.

Functional annotation of transcripts was performed using the Database for Annotation, Visualization, and Integrated Discovery (DAVID) [39] and the Bibliosphere pathway edition (Genomatix).

### Quantitative real-time RT-PCR

Amplifications of 50 ng cDNA were performed with an ABI7900HT machine (Applied Biosystems) in triplicates in 10 μl reaction mixtures containing 1 × TaqMan Universal PCR Master Mix (Applied Biosystems), 200 nM of primers and 0.25 μl dual-labeled probe (Roche ProbeLibrary). The reaction parameters were as follows: 2-min 50°C hold, 30-min 60°C hold, and 5-min 95°C hold, followed by 45 cycles of 20-s 94°C melt and 1-min 60°C anneal/extension. Measurements were performed in triplicate. Results were analyzed with an ABI sequence detector software version 2.3 using the ΔΔCt method for relative quantitation. Primer sequences and Roche Library Probe numbers are listed in Table 1.

**Table 1:** Primer pairs and Roche library probes for real time qRT-PCR validation

Gene	F-Primer (5'-3')	R-Primer (5'-3')	Roche Library Probe
AA467197	aaatggtggatcctactcaacc	gttgccctccggactttt	17
Blvrb	tcctcggagttctcagcttt	gcaccgtcacctcataacct	81
C3	accttacctcggcaagtttct	ttgtagagctgctggtcagg	76
CD36	ttgaaaagtctcggacattgag	tcagatccgaacacagcgta	6
CD83	gctctcctatgcagtgtcctg	ggatcgtcagggaataggc	2
Cfb	ctcgaacctgcagatccac	tcaaagtcctgcggtcgt	1
Cst7	atgtcagcaaagccctggta	ggtcttcctgcatgtagttcg	67
Cxcl10	gctgccgtcattttctgc	tctcactggcccgtcatc	3
Ddit3	ccaccacacctgaaagcag	tcctcataccaggcttcca	33
Gbp2	tgtagaccaaaagttccagacaga	gataaaggcatctcgcttgg	62
Gbp3	aagattgagctgggctacca	gaaactcttgagaacctcttttgc	73
Gclm	tggagcagctgtatcagtgg	caaaggcagtcaaatctggtg	18
Gusb	gtgggcattgtgctacctg	atttttgtcccggcgaac	25
Hmox1	ctgctagcctggtgcaaga	ccaacaggaagctgagagtga	25
Hp	ccctgggagctgttgtca	ctttgggcagctgtcatctt	15
Hprt1	tcctcctcagaccgctttt	cctggttcatcatcgctaatc	95
Ifi44	ctgattacaaaagaagacatgacagac	aggcaaaaccaaagactcca	78
Ifitm3	aacatgcccagagaggtgtc	accatcttccgatccctagac	84
Ifitm6	ccggatcacattacctggtc	catgtcgcccaccatctt	27
IL-6	gatggatgctaccaaactggat	ccaggtagctatggtactccaga	6
iNos	ctttgccacggacgagac	tcattgtactctgagggctga	13
Irf7	cttcagcactttcttccgaga	tgtagtgtggtgacccttgc	25
Kdr	cagtggtactggcagctagaag	acaagcatacgggcttgttt	68
Lcn2	atgtcacctccatcctggtc	cctgtgcatatttcccagagt	1
Lpcat1	aatgtgaggcgtgtcatgg	ggcagtcctcaaatgtatagtcg	81
Marco	cagagggagagcacttagcag	gccccgacaattcacatt	20
Mpeg1	cacagtgagcctgcacttaca	gcgctttcccaatagcttta	69
Nupr1-F	gatggaatcctggatgaatatga	gtccgacctttccgacct	64
Rnf145	catggacttctggcttctcat	aataaaaagtgttcccagaacctg	67
Saa3	atgctcgggggaactatgat	acagcctctctggcatcg	26
Slpi	gtgaatcctgttcccattcg	cctgagttttgacgcacctc	69
Srxn1	gctatgccacacagagaccata	gtgggaaagctggtgtcct	33
Trib3	gctatcgagccctgcact	acatgctggtgggtaggc	98

### Statistical analysis

Statistical analysis were performed on ΔΔCt data using analysis of variance with a two-sample Student's t test (P < 0.05) unless otherwise indicated. Quantitative data are expressed as mean ± SEM. The levels of gene expression in treated BV-2 cells are shown relative to control cells.

## Results

### Effects of luteolin on selected pro-inflammatory markers

**Figure 1:**
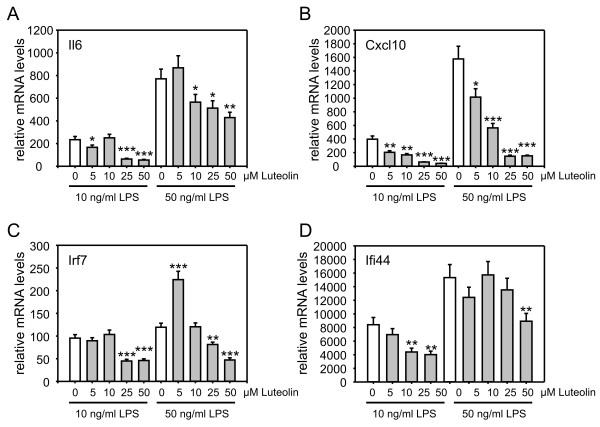
**Dose-dependent suppression of LPS-induced pro-inflammatory gene expression in BV-2 microglia**. BV-2 microglia were treated with the indicated concentrations of luteolin for 1 h and then stimulated with 10 ng/ml or 50 ng/ml LPS for 24 h. Gene expression levels of (A) interleukin 6 (Il6), (B) chemokine (C-X-C motif) ligand 10 (Cxcl10), (C) interferon-regulatory factor 7 (Irf7), and (D) interferon-induced gene 44 (Ifi) were analyzed with real-time qRT-PCR. Expression was normalized to the control gene Gusb and mRNA levels (+/- SEM) are graphed relative to mock-treated control cells. Results are calculated from three independent experiments performed in triplicate measurements. * p ≤ 0.05, ** p ≤ 0.01, and *** p ≤ 0.001 for luteolin + LPS vs. LPS, respectively.

As a basis to study the genome-wide transcriptional effects of luteolin on activated microglia and to validate our cell culture system, we initially performed a dose-response curve for luteolin. Four pro-inflammatory microglia markers with different expression levels and ranges of induction were selected as positive controls for qRT-PCR analyses. Interleukin 6 (Il6) is a well known pro-inflammatory cytokine target of luteolin [34], chemokine (C-X-C motif) ligand 10 (Cxcl10), interferon-regulatory factor 7 (Irf7), and interferon-inducible protein 44 (Ifi44) are LPS-sensitive genes in microglia [36]. BV-2 microglia were pre-treated with different concentrations of luteolin (0, 5, 10, 25, and 50 μM) for 1 h and then stimulated with LPS (10 ng/ml and 50 ng/ml) for a further 24 h period. Neither LPS nor luteolin changed the proliferation rate or cell survival at the concentration levels applied to the cells (data not shown). mRNA levels of Il6 (Fig. 1A), Cxcl10 (Fig. 1B), Irf7 (Fig. 1C), and Ifi44 (Fig. 1D), which were all induced by 10 ng/ml LPS and further increased by 50 ng/ml LPS, were dose-dependently reduced by luteolin (Fig. 1A-D). Luteolin's effects on the four genes were most prominent at 50 μM (Fig. 1A-D) and therefore this concentration was used in all further experiments.

### Luteolin triggers global changes in the microglial transcriptome

**Figure 2:**
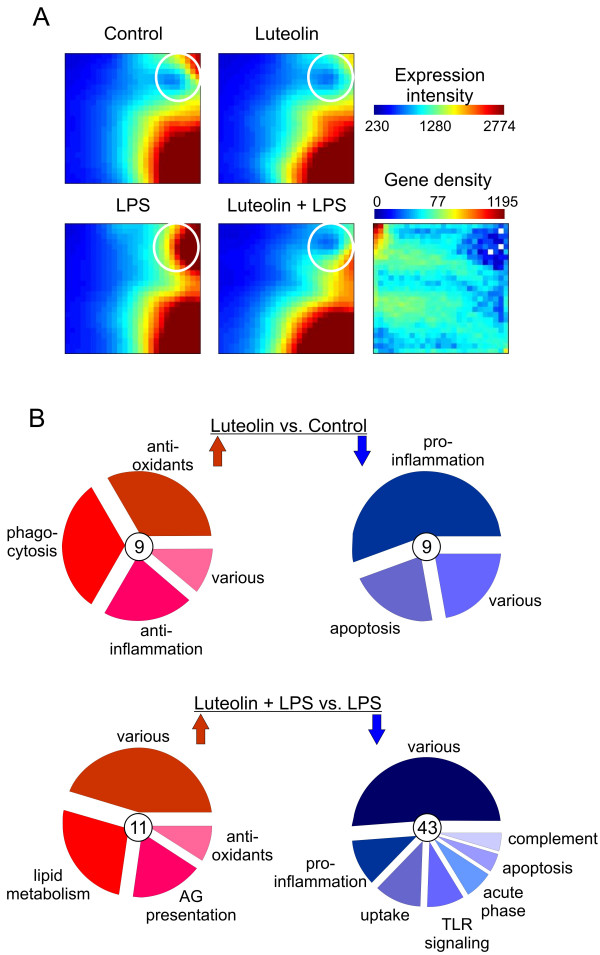
**Luteolin triggers global transcriptomic changes in non-activated and LPS-activated BV-2 microglia**. (A) Comparative Gene Expression Dynamics Inspector (GEDI) analysis of DNA-microarray datasets from control BV-2 cells or cells treated with 50 μM luteolin, 50 ng/ml LPS, or 50 μM luteolin + 50 ng/ml LPS. The white circle denotes the most prominent expression changes in several corresponding gene clusters. (B) Genomatix ChipInspector single probe analysis of differentially expressed transcripts in luteolin versus control-treated cells (top) or luteolin + LPS treated cells versus LPS stimulated cells. Triplicate Affymetrix Mouse 430 2.0 GeneChips were analyzed with a false-discovery-rate (FDR) <0.1%, 3 significant probes and log2 ratios >1. Differentially expressed transcrips from both comparisons were subjected to pathway analysis and clusters with ≥ 3 genes were defined as an independent group. Up-regulated genes are shown on the left side in red and down-regulated genes are displayed on the right side in blue. The total numbers of significantly up- or down-regulated genes from both comparions are indicated in circles placed in the centers of individual pie charts. All differentially expressed genes are listed in Tables 2 and 3, respectively.

Our next goal was to capture and compare the transcriptional profile of non-activated and LPS-activated BV-2 microgli treated each with 50 μM luteolin for 24 h using Affymetrix Mouse Genome 430 2.0 GeneChips. Twelve microarray analyses from three independent stimulation experiments were performed and high stringency criteria with a minimal signal intensity of 50 fluorescence units were used. The complete microarray RMA datasets and all raw data (Affymetrix CEL-files) were stored in the NCBI GEO repository as record GSE18740. We used the Gene Expression Dynamics Inspector (GEDI) to determine the global patterns of gene expression in the four different conditions, untreated, luteolin-treated, LPS-treated, and luteolin + LPS-treated microglia. GEDI is based on self-organizing maps to identify genome-wide transcriptome activity via 'gestalt' recognition [38]. GEDI is sample-oriented rather than gene-oriented, which facilitates the identification of genome-wide patterns. Each mosaic tile in the GEDI map represents a gene group or cluster that is expressed at similar levels, with blue color indicating a low level and red corresponding to high expression. The four GEDI maps clearly show a dynamic regulation of gene expression in stimulated versus non-stimulated microglia (Fig. 2). Especially the upper right positions in the control dataset and more pronounced in the LPS-treated dataset display an inverse regulation of the gene clusters following luteolin stimulation (Fig. 2A, white circles). These results demonstrate that stimulation with luteolin has a major impact on the global pattern of gene expression notably in activated microglia and to a lower extend in resting cells.

We have previously shown that a single probe analysis of Affymetrix microarrays using the Genomatix ChipInspector tool with the Significance Analysis of Microarray (SAM) algorithm improves detection of regulated transcripts [40]. We therefore used ChipInspector to analyze our dataset applying a false discovery rate (FDR) of 0.1%, a minimal probe coverage of 3, and a minimum log2 ratio of 1 (fold change of 2.0). Thereby, 18 significantly regulated genes were identified in luteolin-treated versus non-treated microglia (Table 2) and 54 differentially expressed genes were detected in luteolin + LPS versus LPS-stimulated cells (Table 3). Comparison of the total numer of differentially expressed genes indicated that the effects of luteolin were more pronounced in LPS-activated BV-2 cells than in resting microglia. Also, the overall fold change range was higher in the LPS-treated microglia than in the resting BV-2 cells when stimulated with luteolin. These finding correlate with the gene cluster patterns observed in GEDI analysis and implicate that luteolin is an effective counter-player of pro-inflammatory microglial activation.

**Table 2:** Differentially expressed transcripts after 24 h stimulation of BV-2 cells with 50 μM luteolin

Nr	ID	Symbol	Gene Name	FC*	Cov
**UP-REGULATED**					
1	74315	Rnf145	Ring finger protein 145	2.87	11
2	76650	Srxn1	Sulfiredoxin 1 homolog	2.38	5
3	19252	Dusp1	Dual specificity phosphatase 1	2.35	10
4	12267	C3ar1	Complement component 3a receptor 1	2.30	22
5	12491	Cd36	CD36 antigen	2.28	10
6	12475	Cd14	CD14 antigen	2.25	10
7	233016	Blvrb	Biliverdin reductase B	2.22	9
8	15368	Hmox1	Heme oxygenase 1	2.17	10
9	210992	Lpcat1	Lysophosphatidylcholine acyltransferase 1	2.13	11
**DOWN-REGULATED**					
1	13198	Ddit3	DNA-damage inducible transcript 3	-5.21	11
2	12862	Cox6a2	Cytochrome c oxidase, subunit VI a, polypeptide 2	-2.89	9
3	228775	Trib3	Tribbles homolog 3	-2.62	22
4	56312	Nupr1	Nuclear protein 1	-2.36	15
5	13011	Cst7	Cystatin F	-2.19	9
6	223920	Soat2	Sterol O-acyltransferase 2	-2.16	8
7	16149	Cd74	CD74 antigen	-2.10	8
8	17064	Cd93	CD93 antigen	-2.08	11
9	213002	Ifitm6	Interferon induced transmembrane protein 6	-2.06	8

Significance analysis of triplicate microarrays was performed with a false discovery rate of 0.1% and a minimum probe coverage of 3. ID, Entrez Gene ID; FC: Fold change; Cov: probe coverage; * validated by Taqman realtime RT-PCR

**Table 3:** Differentially expressed transcripts after 24 h stimulation with 50 μM luteolin + 50 ng/ml LPS versus 50 ng/ml LPS

Nr	ID	Symbol	Gene Name	FC*	Cov
**UP-REGULATED**					
1	74315	Rnf145	Ring finger protein 145	3.27	11
2	210992	Lpcat1	Lysophosphatidylcholine acyltransferase 1	2.55	11
3	12522	Cd83	CD83 antigen	2.51	11
4	14630	Gclm	Glutamate-cysteine ligase, modifier subunit	2.46	10
5	16889	Lipa	Lysosomal acid lipase A	2.31	6
6	56336	B4galt5	UDP-Gal:betaGlcNAc beta 1,4-galactosyltransferase, polypeptide 5	2.30	8
7	14950	H13	Histocompatibility 13	2.30	7
8	14104	Fasn	Fatty acid synthase	2.28	4
9	12125	Bcl2l11	BCL2-like 11	2.16	14
10	16542	Kdr	Kinase insert domain protein receptor	2.16	9
11	216345	Ccdc131	Coiled-coil domain containing 131	2.11	7
**DOWN-REGULATED**					
1	66141	Ifitm3	Interferon induced transmembrane protein 3	-13.74	9
2	433470	AA467197	Expressed sequence AA467197, miR-147	-9.32	9
3	16819	Lcn2	Lipocalin 2	-6.87	10
4	14469	Gbp2	Guanylate binding protein 2	-6.41	16
5	14962	Cfb	Complement factor B	-5.94	8
6	13198	Ddit3	DNA-damage inducible transcript 3	-5.58	11
7	16181	Il1rn	Interleukin 1 receptor antagonist	-5.28	33
8	55932	Gbp3	Guanylate binding protein 3	-4.72	10
9	56312	Nupr1	Nuclear protein 1	-4.35	15
10	75345	Slamf7	SLAM family member 7	-4.14	9
11	20210	Saa3	Serum amyloid A 3	-3.78	7
12	17386	Mmp13	Matrix metallopeptidase 13	-3.63	11
13	15439	Hp	Haptoglobin	-3.56	11
14	17167	Marco	Macrophage receptor with collagenous structure	-3.34	8
15	12266	C3	Complement component 3	-3.29	6
16	12870	Cp	Ceruloplasmin	-3.05	5
17	13011	Cst7	Cystatin F	-3.03	9
18	23882	Gadd45g	Growth arrest and DNA-damage-inducible 45 gamma	-2.95	11
19	242341	Atp6v0d2	ATPase, H+ transporting, lysosomal V0 subunit D2	-2.81	13
20	68774	Ms4a6d	Membrane-spanning 4-domains, subfamily A, member 6D	-2.75	19
21	83673	Snhg1	Small nucleolar RNA host gene 1	-2.75	24
22	12494	Cd38	CD38 antigen	-2.73	6
23	19655	Rbmx	RNA binding motif protein, X chromosome	-2.73	11
24	56405	Dusp14	Dual specificity phosphatase 14	-2.68	10
25	213002	Ifitm6	Interferon induced transmembrane protein 6	-2.57	8
26	12517	Cd72	CD72 antigen	-2.55	9
27	14129	Fcgr1	Fc receptor, IgG, high affinity I	-2.55	11
28	14130	Fcgr2b	Fc receptor, IgG, low affinity IIb	-2.48	27
29	231532	Arhgap24	Rho GTPase activating protein 24	-2.46	8
30	29818	Hspb7	Heat shock protein family, member 7 (cardiovascular)	-2.45	5
31	17476	Mpeg1	Macrophage expressed gene 1	-2.43	6
32	66222	Serpinb1a	Serine peptidase inhibitor, clade B, member 1a	-2.41	10
33	78771	Mctp1	Multiple C2 domains, transmembrane 1	-2.39	8
34	20568	Slpi	Secretory leukocyte peptidase inhibitor	-2.39	9
35	12507	Cd5	CD5 antigen	-2.35	9
36	50778	Rgs1	Regulator of G-protein signaling 1	-2.35	11
37	21897	Tlr1	Toll-like receptor 1	-2.20	5
38	16149	Cd74	CD74 antigen	-2.17	5
39	73167	Arhgap8	Rho GTPase activating protein 8	-2.13	8
40	15064	Mr1	Major histocompatibility complex, class I-related	-2.06	7
41	347722	Centg2	Centaurin, gamma 2	-2.04	11
42	98365	Slamf9	SLAM family member 9	-2.04	8
43	72999	Insig2	Insulin induced gene 2	-2.00	8

Significance analysis of triplicate microarrays was performed with a false discovery rate of 0.1% and a minimum probe coverage of 3. ID, Entrez Gene ID; FC: Fold change; Cov: probe coverage; * validated by Taqman realtime RT-PCR

### Luteolin regulates important immune pathways and changes the microglial transcriptional phenotype

Our next aim was to put the newly identified luteolin-regulated expression patterns into a specific biological context. We used all differentially expressed genes to perform a classification into functional categories with the Database for Annotation, Visualization, and Integrated Discovery (DAVID). The major enriched functional categories in luteolin-treated resting microglia were *anti-oxidants*, *phagocytosis*, and *anti-inflammation *for up-regulated genes and *pro-inflammation *and *apoptosis *for down-regulated genes (Fig. 2B, upper part). These pathways indicate that luteolin stimulated anti-oxidant and anti-inflammatory transcriptional programs which could potentially reflect M2 macrophage polarization. Moreover, basal pro-inflammatory and pro-apoptotic gene expression was blocked in non-activated BV-2 microglia. In luteolin + LPS co-treated versus LPS-treated cells, 11 induced transcripts represented pathways of *lipid metabolism*, *antigen presentation*, and *anti-oxidants *(Fig. 2B, bottom left). Interestingly, a large number of down-regulated genes covered the pathway categories *pro-inflammation*, *toll-like receptor *(TLR) *signaling*, *acute phase response*, *apoptosis*, and *complement factors *(Fig. 2B, bottom right). These results implicate that luteolin-induced transcriptomic changes promote a stable anti-inflammatory, anti-oxidant, and anti-apoptotic microglial phenotype reminescent of M2 macrophages. Our microarray data also identified a significant number of genes that could not be grouped into larger immune-related pathways and that have not been previously connected to microglial activation or flavonoid stimulation (Tables 2 and 3).

### Microarray validation by qRT-PCR confirms novel luteolin-regulated target genes

**Figure 3:**
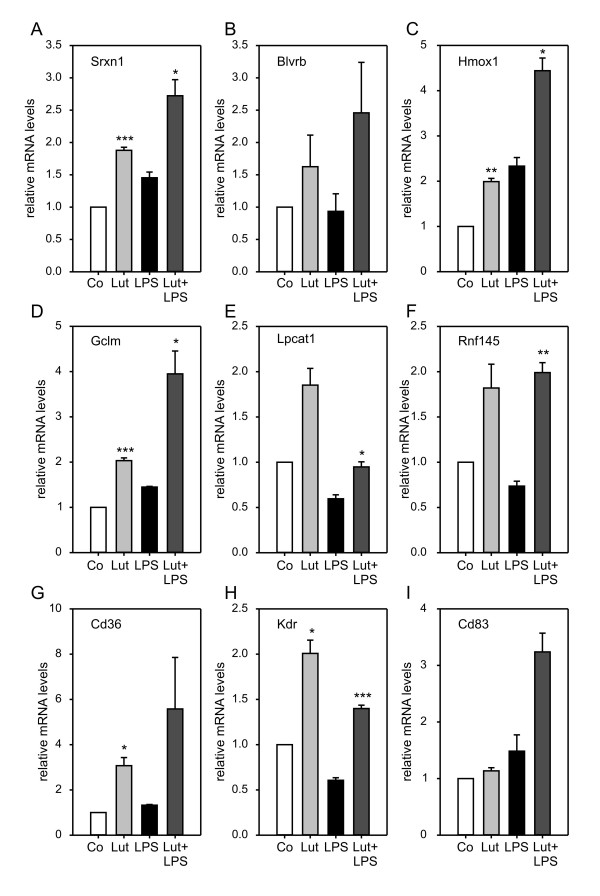
**Luteolin induces anti-oxidant, anti-inflammtory, and survival pathways**. Real-time qRT-PCR validation of transcripts in BV-2 microglia stimulated with 50 μM luteolin, 50 ng/ml LPS, or 50 μM luteolin + 50 ng/ml LPS. Relative mRNA levels were quantified for (A) Sulfiredoxin 1 (Srxn1), (B) Biliverdin reductase b (Blvrb), (C) Heme oxigenase 1 (Hmox1), (D) Glutamate-cysteine ligase modifier subunit (Gclm), (E) Lysophosphatidylcholine acyltransferase 1 (Lpcat1), (F) Ring finger protein 145 (Rnf145), (G) Cd36 antigen (Cd36), (H) Kinase insert domain protein receptor (Kdr), and (I) Cd83 antigen (Cd83). Expression was normalized to the control gene Gusb and mRNA levels (+/- SEM) are graphed relative to mock-treated control cells. Results are calculated from three independent experiments performed in triplicate measurements. * p ≤ 0.05, ** p ≤ 0.01, *** p ≤ 0.001 for luteolin vs. control and luteolin + LPS vs. LPS, respectively.

To validate a significant portion of the DNA microarray results, real-time qRT-PCR analyses were carried out with RNA samples from three independent BV-2 replicate experiments. We focused on representative candidates from different biological pathway which have not been previously described as flavonoid targets. 22 genes fulfilled these criteria and were analzyed with qRT-PCR. In the first set of experiments, mRNA levels of genes up-regulated by luteolin treatment were assessed (Fig. 3). Transcripts of Sulfirexodin 1 (Srxn1), Biliverdin reductase B (Blvrb), Heme oxigenase 1 (Hmox1), and Glutamate-cysteine ligase (Gclm) are components of the cellular anti-oxidant response [41-43] and were all induced by luteolin treatment (Fig. 3A-D). We could further validate increased levels of Lysophosphatidylcholine acyltransferase (Lpcat1), Ring finger protein 145 (Rnf145), Cd36 antigen, Kinase insert domain receptor (Kdr, alias Vegfr2), and Cd83 antigen. Lpcat1 catalyzes the inactivation of inflammatory lipids [44], whereas Cd36 and Kdr are surface receptors required for phagocytic uptake and chemotactic migration, respectively [45,46]. The function of Rnf145 is currently unknown.

**Figure 4:**
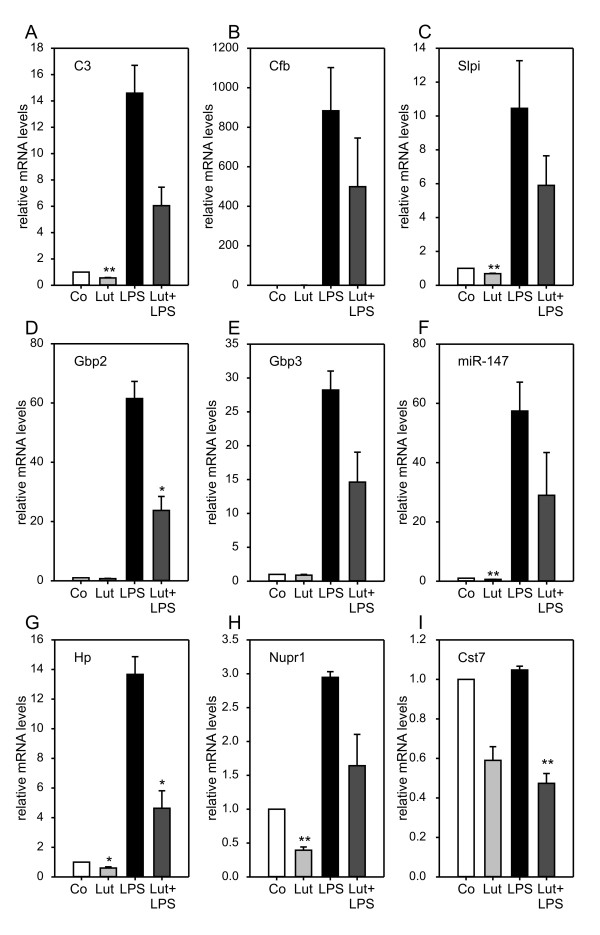
**Luteolin inhibits pro-inflammtory and stress-related pathways**. Real-time qRT-PCR validation of transcripts in BV-2 microglia stimulated with 50 μM luteolin, 50 ng/ml LPS, or 50 μM luteolin + 50 ng/ml LPS. Relative mRNA levels were quantified for (A) Complement component 3 (C3), (B) Complement factor B (Cfb), (C) Serine leukocyte peptidase inhibitor (Spli), (D) Guanylate binding protein 2 (Gnbp2), (E) Guanylate binding protein 3 (Gnbp3), (F) micro RNA 147 (miR-147), (G) Haptoglobin (Hp), (H) Nuclear protein 1 (Nupr1), and (I) Cystatin F (Cst7). Expression was normalized to the control gene Gusb and mRNA levels (+/- SEM) are graphed relative to mock-treated control cells. Results are calculated from three independent experiments performed in triplicate measurements. * p ≤ 0.05, ** p ≤ 0.01 for luteolin vs. control and luteolin + LPS vs. LPS, respectively.

In the next set of qRT-PCR experiments, down-regulated transcripts involved in pro-inflammatory activation and acute phase response were analyzed. Complement factor C3 (C3), Complement factor b (Cfb), Secreted leukocyte peptidase inhibitor (Slpi), and the pro-inflammatory Guanylate binding proteins 2 (Gbp2) and Gbp3 [47] showed diminished mRNA levels in luteolin-treated samples (Fig. 4A-D). The same tendency was seen in the newly identified microRNA miR-147, which is involved in toll-like receptor signaling [48], the acute phase gene Haptoglobin (Hp), the stress-related gene Nuclear protein 1 (Nupr1, alias p8) [49], and Cystatin 7 (Cst7) (Fig. 4F-I).

**Figure 5:**
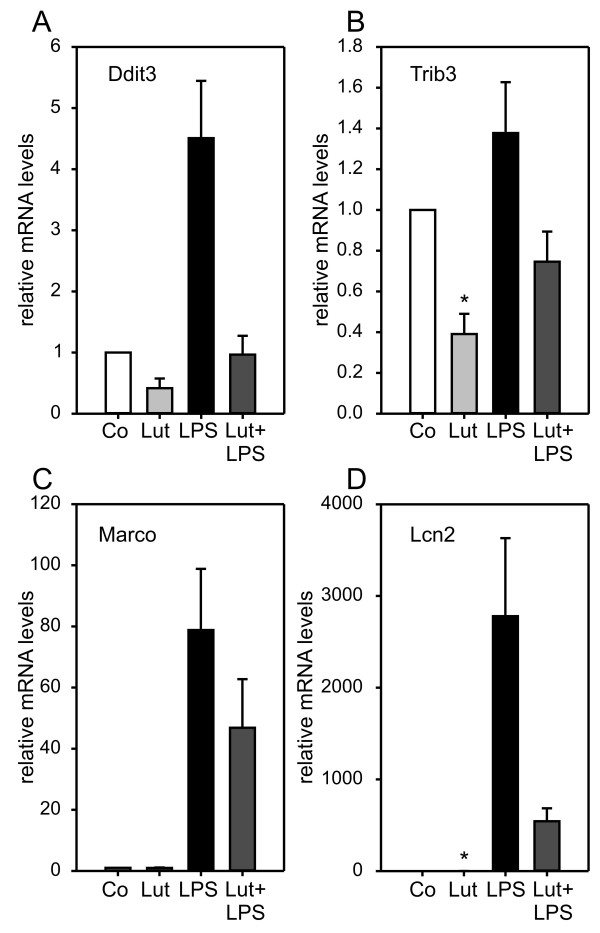
**Luteolin inhibits apoptosis-related pathways and reduces de-ramification genes**. Real-time qRT-PCR validation of transcripts in BV-2 microglia stimulated with 50 μM luteolin, 50 ng/ml LPS, or 50 μM luteolin + 50 ng/ml LPS. Relative mRNA levels were quantified for (A) DNA-damage inducible transcript 3 (Ddit3), (B) Tribbles homolog 3 (Trib3), (C) Macrophage receptor with collagenous structure (Marco), and (D) lipocalin 2 (Lcn2). Expression was normalized to the control gene Gusb and mRNA levels (+/- SEM) are graphed relative to mock-treated control cells. Results are calculated from three independent experiments performed in triplicate measurements. * p ≤ 0.05 for luteolin vs. control and luteolin + LPS vs. LPS, respectively.

As third group of genes validated by qRT-PCR, four luteolin-repressed genes related to apoptosis and microglial shape formation were studied (Fig. 5A-D). The apoptotic mediators DNA-damage inducible transcript 3 (Ddit3, alias Chop, Gadd153) and Tribbles homolog 3 [50,51] were down-regulated by luteolin in non-activated as well as LPS-activated microglia (Fig. 5A, B), indicating anti-apoptotic protection mechanisms elicited by luteolin. In line with these findings, we also observed reduced expression of the Macrophage receptor with collagenous structure (Marco) and Lipocalin 2 (Lcn2), two molecules with dual roles in apoptosis and de-ramification of activated microglia [52,53].

### Luteolin changes microglial morphology and inhibits NO secretion

**Figure 6:**
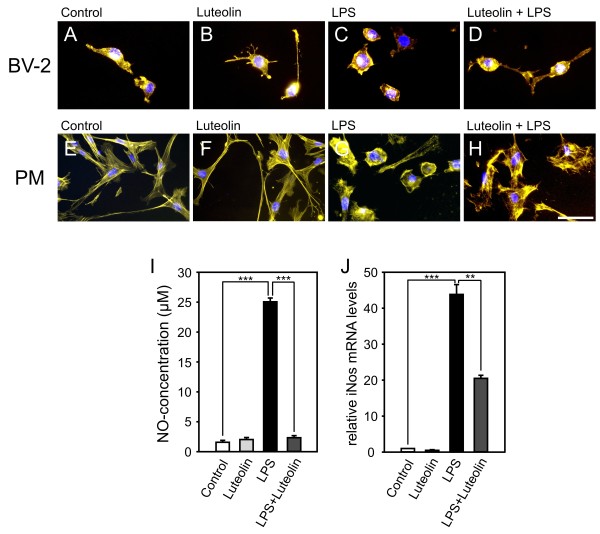
**Luteolin promotes ramification of microglia and inhibits NO-synthesis**. Effects of luteolin, LPS and Luteolin + LPS on BV-2 (A-D) and primary brain microglia (E-H) cell morphology and actin cytoskeleton. Phallodin-TRITC-staining of F-actin bundles and DAPI costaining reveals that 24 h treatment with 50 μM luteolin in non-activated (B, F) or LPS-activated microglia (D, H) supports ramification. (I) NO release from BV-2 cells treated for 24 h with 50 μM luteolin, 50 ng/ml LPS, or LPS + luteolin. The micrographs and data shown are from one representative experiment out of three independent experiments with the same tendencies. Scale bar, 50 μM. (J) Real-time qRT-PCR analysis of iNos transcripts in BV-2 microglia stimulated with 50 μM luteolin, 50 ng/ml LPS, or 50 μM luteolin + 50 ng/ml LPS. Expression was normalized to the control gene Gusb and mRNA levels (+/- SEM) are graphed relative to mock-treated control cells. Results are calculated from two independent experiments performed in triplicate measurements. *** p ≤ 0.001, ** p ≤ 0.01 for LPS vs. control and luteolin + LPS vs. LPS, respectively.

To assess whether the particular gene expression profiles measured in luteolin-stimulated microglia translate into detectable functional properties, phenotypic characterization was performed. The general activation state and morphological phenotype of microglia is particularly reflected by their cell shape and cytoskeletal organization. To detected morphological changes and for visualization of filopodia we performed phalloidin staining. Conventional BV-2 microglia cultures were low level activated cells with a flat shape and some filopodia (Fig. 6A). Culture of BV-2 cells in the presence of luteolin lead to considerable ramification and formation of long protrusions (Fig. 6B), indicating induction of a surveillance state. LPS-activation of BV-2 cells caused formation of a round cell shape with retracted filopodia (Fig. 6C). In contrast, co-incubation of LPS-treated cells with luteolin sustained the ramified microglial morphology (Fig. 6D). A similar effect was seen in primary mouse microglia, where luteolin increased the length of filopodia in non-activated cells (Fig. 6E, F) and caused flatening and ramification of LPS-treated cells (Fig. 6G, H). To test the direct effect of luteolin on microglial secretion of toxic metabolites, NO levels were determined in the supernatant of BV-2 cells. Treatment of BV-2 cells with luteolin alone did not result in increased NO concentrations (Fig. 6I), whereas stimulation with LPS markedly increased secreted NO levels. Co-incubation of LPS-activated cells with luteolin nearly completely abolished NO secretion (Fig. 6I). These data indicate that luteolin favors the ramified surveillance state of microglia and effectively inhibits cytotoxic NO formation. Kim *et al*. previously reported that luteolin triggered a blockade of NO secretion in LPS-stimulated BV-2 microglia, which was mediated by inhibition of inducible NO synthase (iNos) protein expression [54]. To indenpendently verify these data by qRT-PCR, we analyzed iNos mRNA levels in BV-2 cells treated with luteolin, LPS, or both simultaneously. LPS caused a more than 40-fold increase in iNos transcripts and luteolin co-treatment significantly reduced iNos levels more than 2-fold. Thus, our expression data corroborate the findings by Kim *et al*. and provide a reasonable explanation for reduced NO secretion levels.

**Figure 7:**
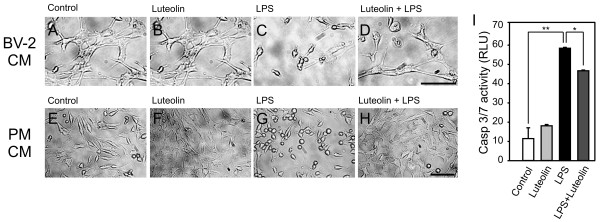
**Luteolin inhibits microglial neurotoxicity on photoreceptors**. Phase contrast micrographs showing morphological changes of 661W photoreceptor cultures treated with conditioned media from BV-2 cells (A-D) or primary brain microglia (E-H). The supernatant from control-stimulated (A, E), 50 μM luteolin-treated (B, F), 50 ng/ml LPS-treated (C, G), or 50 μM luteolin + 50 ng/ml LPS-treated cells (D, H) was added to 661W photoreceptor cells, respectively. The micrographs shown are from one representative experiment out of three independent experiments with the same tendencies. Scale bar, 50 μM. (I) Apoptosis-related caspase 3/7 activation in 661W photoreceptor cells incubated with conditioned media from control-stimulated, 50 μM luteolin-treated, 50 ng/ml LPS-treated, or 50 μM luteolin + 50 ng/ml LPS-treated BV-2 cells. Results are calculated from two independent experiments performed in triplicate measurements. ** p ≤ 0.01, * p ≤ 0.05 for LPS vs. control and luteolin + LPS vs. LPS, respectively.

To test the hypothesis that luteolin leads to decreased microglial neurotoxicity, a culture system of 661W photoreceptor cells with conditioned medium (CM) from BV-2 cells or primary microglia was established. 661W, a retina-derived cell line [55], represents a valuable model to study microglial neurotoxicity in the special context of retinal degeneration [36]. 661W cells were incubated for 24 h with culture supernatants from unstimulated, luteolin-, LPS- or LPS + luteolin-treated cells and 661W photoreceptor cell morphology was assessed by phase contrast microscopy. 661W cells in their own medium grew confluent after 24 h and the presence of CM from control- or luteolin-treated BV-2 cells did not affect cell morphology (Fig. 7A, B). As described previously [56], confluent 661W cells flattened out and often multiple cells were connected to each other through their projected extensions (Fig. 7A, B). In contrast, 661W cells co-incubated with LPS-stimulated BV-2 supernatant appeared elongated and smaller, leading to prominent cell-free areas present in the culture (Fig. 7C). When adding CM from LPS + luteolin-stimulated BV-2 cells, nearly normal cell characteristics were retained (Fig. 7D). Similar morphological changes of 661W cells were observed when cultured in the presence of primary microglia CM (Fig. 7E-H). Direct incubation of photoreceptor cells with LPS, luteolin, or both had no effects on cell morphology (data not shown), indicating that the observed changes in 661W cell growth arise from secreted microglial compounds.

To correlate the microscopic findings with functional data, we next studied the influence of microglia-secreted products on caspase-related apoptotic signaling in the neuronal cell model. 661W cells cultured in the presence of CM from LPS-stimulated BV-2 cells displayed a strong induction of caspase 3/7 activity (Fig. 7I). When CM from microglia co-treated with LPS + luteolin was used, 661W apoptosis was still present but was significantly diminished (Fig. 7I). Culture media from BV-2 cells kept in the presence of luteolin alone had no effect on 661W apoptosis. These findings implicate that luteolin either stimulates microglia to produce less pro-apoptotic substances or actively promotes the release of survival signals.

## Discussion

Like other plant-derived flavonoids, luteolin has a variety of biological activities including well-known anti-mutagenic and anti-tumorigenic properties [57]. Moreover, this flavone possesses direct anti-oxidant activity, which is attributed to structural features of all flavonoids, which favor scavenging of reactive oxygen and nitrogen species [58]. Although the anti-oxidant and anti-inflammatory activities of luteolin may be also useful in the treatment of many chronic inflammatory diseases including neurodegeneration, only little information is available about luteolin-mediated transcriptional mechanisms or molecular targets in microglia [59].

We have therefore performed the first genome-wide study of luteolin-mediated transcriptional effects in microglia. To our surprise, luteolin treatment did not only change expression levels of a few transcripts but had a broad and strong impact on the transcriptome of resting and particularly of LPS-activated microglia. The microarray dataset and the qRT-PCR validations revealed several luteolin-regulated pathways. Luteolin caused simultaneous up-regulation of four important anti-oxidant enzymes Srxn1, Blvrb, Gclm, and Hmox1. These data are consistent with earlier findings demonstrating increased Hmox1 transcription in RAW264.7 macrophages after luteolin treatment [60]. Stimulation with the flavonoid induced binding of the transcription factor NF-E2-related factor 2 (Nrf2) to anti-oxidant response elements (ARE) in the Hmox1 promoter region [60]. Luteolin is a potent activator of Nrf2 [61] and the majority of anti-oxidant enzymes contain ARE in their regulatory regions, including Srxn1 [62]. Moreover, mouse embryonic fibroblasts derived from Nrf2 -/- mice showed significantly lower Blvrb and Gclm mRNA levels upon Diquat induction [63]. Therefore, we speculate that increased microglial expression of Srxn1, Blvrb, and Gclm is also mediated by activation of Nrf2. This hypothesis is further corroborated by the protective functions of Nrf2 in several microglia-related neurodegenerative disorders [64].

Luteolin significantly enhanced mRNA synthesis of five other genes involved in different biological pathways. Lpcat1 is a lysophospholipid acyltransferase implicated in anti-inflammatory responses by converting lyso-platelet activation factor (lyso-PAF) to PAF and lyso-phosphatidylcholine (lyso-PC) to PC [65]. LPC exerts considerable neuro-inflammatory reactivity in the brain and inhibition of LPC signaling in astrocytes and microglia confers neuroprotection [66]. Lpcat1 is also highly expressed in the retina [44], indicating that luteolin-induced Lpact1 levels could lead to diminished LPC levels in retinal microglia. Rnf145 was also up-regulated by luteolin but the function of this protein remains to be determined. Cd36 and Kdr (alias Vegfr2) were also significantly induced by luteolin in non-activated as well as activated microglia. The pattern recognition receptor Cd36 signals to the actin cytoskeleton and regulates microglial migration and phagocytosis [67], whereas Kdr is involved in the chemotactic response of microglia [46]. We thus speculate that luteolin-mediated expression of both genes could result in increased phagocytic responses of microglia without inducing inflammation.

Several reports have demonstrated that luteolin inhibits pro-inflammatory cytokine expression in various cell types by blocking NFkB (reviewed in [28]). Our microarray data confirmed these findings and revealed further NFkB target genes including the recently discovered microRNA miR-147 [48]. Recently, Jang et al. showed that luteolin reduced Il6 production mainly by inhibiting JNK signaling and AP1 activation [34]. Luteolin did not affect IkB-α degradation or NFkB DNA binding in brain microglia, implicating that luteolin-mediated effects in microglia are not solely dependent on NFkB blockade [34]. In line with this notion, our luteolin-regulated expression profiles identified several genes with NFkB-independent promoter control. Likewise, luteolin down-regulated complement factor 3, which is regulated by AP1 [68] and blocked expression of Slpi, which is a target of interferon regulatory factor 1 (IRF1) [69]. Luteolin also diminished mRNA levels of the pro-inflammatory GTPase Gbp2 and the acute phase protein Haptoglobin, which are both regulated by signal transducers and activators of transcription (STATs) [70,71]. These data clearly show that luteolin dampens microglia activation by interfering with several divergent signaling pathways.

The luteolin-regulated differential expression patterns also revealed genes involved in microglial apoptosis and ramification. Microglia are more susceptible than macrophages to apoptosis [72] and recent evidence indicates that microglial apoptosis and senescence may precede neurodegeneration [73]. Ddit 3 and Trib3, which were both induced by LPS and suppressed by luteolin, support stress and NO-mediated apoptosis [50,51]. We therefore hypothesize that luteolin could promote the survival of activated and stressed microglia in an environment of early neurodegeneration. Our expression data also revealed the unexpected finding that luteolin down-regulated Lcn2 and Marco, two molecules involved in microglial ramification and formation of filopodia. Lee *et al*. demonstrated that stable expression of Lcn in BV-2 microglia, the same cell line we used in our experiments, induced a round cell shape with a loss of processes [52]. In line with this, over-expression of the scavenger receptor Marco in dendritic cells caused rounding of cells and down-regulated antigen uptake [53]. Thus, we hypothesized that the observed changes in mRNA levels of both genes might also translate into different morphological characters.

The morphological and functional assays fully supported the implications from gene expression profiles and revealed a direct effect of luteolin on the microglial phenotype. Luteolin stimulated the formation of filopodia and caused ramification of BV-2 cells and primary microglia even in the setting of strong LPS activation. Moreover, NO secretion was completely blocked in LPS-activated microglia upon co-incubation with luteolin. We studied the effects of conditioned media from microglia on cultured photoreceptor-like 661W cells and demonstrated that luteolin-treatment effectively protected 661W cells from LPS-induced microglial toxicity. Since NO and other reactive oxygen species are the major radicals secreted from microglia, we speculate that luteolin directly inhibits the secretion of these cytotoxic radicals. Our hypothesis is corroborated by recent data demonstrating that luteolin concentration-dependently attenuated LPS-induced dopaminergic neuron loss by blocking NO secretion from cultured rat microglia [32].

## Conclusions

We have shown that the flavonoid luteolin is a potent modulator of microglial activation, cell shape, and effector functions. Luteolin induced global changes in the transcriptome of resting or LPS-activated microglia leading to a polarized M2-like phenotype with anti-inflammatory and neuroprotective characteristics. Luteolin's mechanisms of action appear to target several independent pathways independent of NFkB. Our results provide a basis to develop immuno-modulatory and neuroprotective therapies for the treatment of neurodegenerative disoders.

## Competing interests

The authors declare that they have no competing interests.

## Authors' contributions

KD, SE, and DK carried out all cell cultures and qRT-PCR experiments. MK, JH, and YW analyzed qRT-PCR and functional data. CM performed microarray hybridizations and raw data analyses. RF analyzed microarray data and critically read the manuscript. TL designed the study, obtained funding, carried out biostatistical analyses of microarrays and wrote the manuscript. All authors read and approved the final manuscript.
