# Correction: Luteolin triggers global changes in the microglial transcriptome leading to a unique anti-inflammatory and neuroprotective phenotype

**DOI:** 10.1186/1742-2094-9-118

**Published:** 2012-06-06

**Authors:** Konstantin Dirscherl, Marcus Karlstetter, Stefanie Ebert, Dominik Kraus, Julia Hlawatsch, Yana Walczak, Christoph Moehle, Rudolf Fuchshofer, Thomas Langmann

**Affiliations:** 1 Institute of Human Genetics, University of Regensburg, Franz-Josef-Strauss-Allee 11, 93053, Regensburg, Germany; 2 Center of Excellence for Fluorescent Bioanalytics, University of Regensburg, Josef-Engert-Str. 9, 93053, Regensburg, Germany; 3 Institute of Human Anatomy and Embryology, University of Regensburg, Universitätsstr. 31, 93053, Regensburg, Germany; 4 Center of Opthalmology, Department of Experimental Immunology of the Eye, University of Cologne, Kerpener Strasse 62, 50925, Cologne, Germany

## Abstract

Correction to Dirscherl K, Karlstetter M, Ebert S, Kraus D, Hlawatsch J, Walczak Y, Moehle C, Fuchshofer R, Langmann T. Luteolin triggers global changes in the microglial transcriptome leading to a unique anti-inflammatory and neuroprotective phenotype. *J Neuroinflammation* 2010, **7**:3.

## Correction

The authors observed that the original study [1] contains an error in Figure seven B (Figure 1B), which depicts the same micrograph as shown in Figure seven A (Figure 1A). All other information is accurate, and this does not affect the findings of the paper.

**Figure 1:**
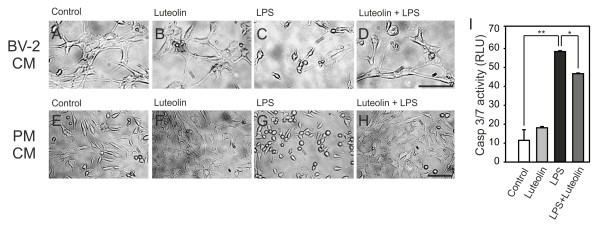


## References

[B1] Dirscherl K Karlstetter M Ebert S Kraus D Hlawatsch J Walczak Y Moehle C Fuchshofer R Langmann T Luteolin triggers global changes in the microglial transcriptome leading to a unique anti-inflammatory and neuroprotective phenotype J Neuroinflammation 2010 7 3 10.1186/1742-2094-7-3 20074346 PMC2819254

